# A Novel Psittacine Adenovirus Identified During an Outbreak of Avian Chlamydiosis and Human Psittacosis: Zoonosis Associated with Virus-Bacterium Coinfection in Birds

**DOI:** 10.1371/journal.pntd.0003318

**Published:** 2014-12-04

**Authors:** Kelvin K. W. To, Herman Tse, Wan-Mui Chan, Garnet K. Y. Choi, Anna J. X. Zhang, Siddharth Sridhar, Sally C. Y. Wong, Jasper F. W. Chan, Andy S. F. Chan, Patrick C. Y. Woo, Susanna K. P. Lau, Janice Y. C. Lo, Kwok-Hung Chan, Vincent C. C. Cheng, Kwok-Yung Yuen

**Affiliations:** 1 State Key Laboratory of Emerging Infectious Diseases, University of Hong Kong, Hong Kong, China; 2 Research Centre of Infection and Immunology, University of Hong Kong, Hong Kong, China; 3 Department of Microbiology, University of Hong Kong, Hong Kong, China; 4 Centre for Health Protection, Department of Health, Hong Kong, China; University of California San Diego School of Medicine, United States of America

## Abstract

*Chlamydophila psittaci* is found worldwide, but is particularly common among psittacine birds in tropical and subtropical regions. While investigating a human psittacosis outbreak that was associated with avian chlamydiosis in Hong Kong, we identified a novel adenovirus in epidemiologically linked Mealy Parrots, which was not present in healthy birds unrelated to the outbreak or in other animals. The novel adenovirus (tentatively named Psittacine adenovirus HKU1) was most closely related to Duck adenovirus A in the *Atadenovirus* genus. Sequencing showed that the Psittacine adenovirus HKU1 genome consists of 31,735 nucleotides. Comparative genome analysis showed that the Psittacine adenovirus HKU1 genome contains 23 open reading frames (ORFs) with sequence similarity to known adenoviral genes, and six additional ORFs at the 3′ end of the genome. Similar to Duck adenovirus A, the novel adenovirus lacks LH1, LH2 and LH3, which distinguishes it from other viruses in the *Atadenovirus* genus. Notably, fiber-2 protein, which is present in *Aviadenovirus* but not *Atadenovirus*, is also present in Psittacine adenovirus HKU1. Psittacine adenovirus HKU1 had pairwise amino acid sequence identities of 50.3–54.0% for the DNA polymerase, 64.6–70.7% for the penton protein, and 66.1–74.0% for the hexon protein with other *Atadenovirus*. The *C. psittaci* bacterial load was positively correlated with adenovirus viral load in the lung. Immunostaining for fiber protein expression was positive in lung and liver tissue cells of affected parrots, confirming active viral replication. No other viruses were found. This is the first documentation of an adenovirus-*C. psittaci* co-infection in an avian species that was associated with a human outbreak of psittacosis. Viral-bacterial co-infection often increases disease severity in both humans and animals. The role of viral-bacterial co-infection in animal-to-human transmission of infectious agents has not received sufficient attention and should be emphasized in the investigation of disease outbreaks in human and animals.

## Author Summary

*Chlamydophila psittaci* is a bacterial pathogen which can cause avian chlamydiosis and human psittacosis. Although *C. psittaci* is frequently detected in birds from the tropical and subtropical regions, large outbreaks of human infections rarely occur. During the investigation of a human psittacosis outbreak that was associated with avian chlamydiosis in Hong Kong, we identified a novel adenovirus in epidemiologically linked Mealy Parrots, which was not present in healthy birds unrelated to the outbreak or in other animals. Since there was a positive correlation between the adenovirus viral load and *C. psittaci* bacterial load in the lungs of the infected Mealy parrots and because adenoviruses are known to cause immunosuppression in birds, we speculate that our novel Psittacine adenovirus may have caused immunosuppression among infected parrots resulting in a larger number of Mealy Parrots being infected by *C. psittaci* with a higher bacterial load, leading to a greater chance of zoonotic transmission to humans.

## Introduction

About 70% of microbial agents causing outbreaks of emerging infectious diseases in humans originate directly from animals [1]. Among respiratory virus infections, the influenza A viruses H5N1 and H7N9 from avian species, and the severe acute respiratory syndrome coronavirus from bats have caused large epidemics [1]–[3]. Atypical bacterial pathogens causing community-acquired pneumonia include *Chlamydophila psittaci* from psittacine birds and *Coxiella burnetti* from livestock and other animals [4]. However, human outbreaks due to zoonotic bacteria associated with the emergence of a novel animal virus in the animal host were not previously documented.

In November 2012, an outbreak of human psittacosis affecting six staff members occurred at the New Territories North Animal Management Centre (NTNAMC) in Hong Kong [5]. The human outbreak was preceded by an outbreak of avian chlamydiosis among the detained Mealy Parrots (*Amazona farinose*). Although birds in the tropical and sub-tropical areas are commonly infected with *C. psittaci*, most infected birds are asymptomatic [6], [7]. Large human outbreaks are rare even among bird handlers [8]. Although co-infection of *C. psittaci* and viruses has been reported in outbreaks of avian species [9]–[12], no virus-bacterium co-infection of implicated avian species has ever been reported in outbreaks of human psittacosis. In this study, we sought to investigate viruses that cause avian co-infection, which may have led to this outbreak of psittacosis.

## Methods

### Outbreak investigation

A case was defined as a staff member working at the NTNAMC who was hospitalized for respiratory tract infection between November 1 and November 30, 2012, and confirmed to have *C. psittaci* infection by polymerase chain reaction (PCR) and/or a four-fold rise in serum microimmunofluorescent antibody titer against *C. psittaci* (Focus Diagnostics, Cypress, California, USA).

### Post-mortem examination of Mealy Parrots and animal samples obtained for microbiological testing

For the investigation of the suspected outbreak of avian chlamydiosis, four Mealy Parrot carcasses from the batch were sent to Tai Lung Veterinary Laboratory of the Agriculture, Fisheries and Conservation Department for postmortem examination and disease diagnosis. Various tissues were collected and tested for *C. psittaci* PCR, bacterial culture and histopathological examination. Postmortem examination was performed with tissue blocks taken for sectioning and staining by hematoxylin and eosin stain. For microbiological testing, tissue (approximately 5 mm×5 mm block) or cloacal swab samples were immersed in viral transport medium [13]. Precautions were taken to avoid cross contamination as described previously [14]. Cloacal swabs, pharyngeal swabs or EDTA blood samples were also obtained from healthy parrots in NTNAMC as controls. Fecal samples were collected from other animals in NTNAMC by cloacal/rectal swabs.

### Total nucleic acid extraction

Total nucleic acid extraction was performed by EZ1 virus Mini Kit v2.0 (Qiagen, Hilden, Germany) according to the manufacturer's instructions. Avian lung, liver, spleen, kidney, and muscle tissues were disrupted mechanically before proceeding to nucleic acid extraction.

### PCR detection of *C. psittaci*

The bacterial load of *C. psittaci* was detected using real-time PCR with LightCycler 96 system (Roche Applied Science, Mannheim, Germany). Primer and probe sequences are presented in Table 1. The lower detection limit was 100 copies per reaction (12,000 copies/ml of viral transport medium).

**Table 1:** Sequences of *C. psittaci* and adenovirus primers and probes used in the study.

Pathogen	Type of PCR	Target gene	Target length (base pairs)	Primer/probe sequences
*Chlamydophila psittaci*	Real-time qPCR	ITS	145	Forward 5′-TTGGTCTGTAAATTATTGATCC-3′
				Reverse 5′-CATTTAGTTTACGATCAAGTATG-3′
				Probe 5′-FAM-ATGCAAGTTAACWTCACCTAAAGACAT-BHQ1-3′
Adenovirus*	Conventional PCR	pol	324	Forward 5′-GTCTACGAYATHTGYGGMATGTA-3′
				Reverse 5′-TCTCCATCCTCDRTTRTGVA-3′
Psittacine adenovirus HKU1	Conventional PCR	pol	175	Forward 5′-GGGTGACTATGTCTATCGACG-3′
				Reverse 5′-GATTTCATACTTCGACAGCG-3′
Psittacine adenovirus HKU1	Real-time qPCR	pol	175	Forward 5′-GGGTGACTATGTCTATCGACG-3′
				Reverse 5′-GATTTCATACTTCGACAGCG-3′
				Probe 5′-FAM-AGGTAGTGTGTCGAGGCGCT-BHQ1-3′

qPCR, quantitative polymerase chain reaction.

*Consensus primers for adenovirus were designed by performing multiple alignments of pol genes of adenovirus and related sequences available in NCBI GenBank.

### Screening and quantification of adenovirus in Mealy Parrots

Tissue and cloacal swab samples of the Mealy Parrots were screened by consensus primers for common respiratory viral agents including influenza virus, coronavirus, infectious bursal disease virus, beak and feather disease virus, hepatitis E virus and the virus families *Paramyxoviridae*, *Picornaviridae*, *Caliciviridae*, *Astroviridae*, *Arteriviridae*, and *Herpesviridae* as previously reported [15]–[18]. For adenovirus, we used a nested PCR as reported previously [19] and a single-step consensus PCR using primers designed by performing multiple alignments of pol genes of adenovirus and related sequences available in NCBI GenBank (Table 1). The PCR products were gel purified using the QIAquick gel extraction kit (Qiagen, Hilden, Germany). Both strands of the PCR products were sequenced twice with an ABI Prism 3130*xl* DNA analyzer (Applied Biosystems, Foster City, California), using the PCR primers. The sequences of the PCR products were compared with known sequences by BLAST analysis against the NCBI database. For quantification of Psittacine adenovirus HKU1, real-time quantitative PCR (qPCR) with specific primers was performed using LightCycler 96 system (Roche Applied Science, Mannheim, Germany) (Table 1). Standards were included for quantification and no false-positive results were observed in the negative controls. The lower limit of detection was 10 copies per reaction (1200 copies/ml of viral transport medium).

### Screening of Psittacine adenovirus HKU1 in other animals and humans

Conventional PCR using specific primers were used to screen for the novel adenovirus (Psittacine adenovirus HKU1) in other animals and humans (Table 1).

### Virus genome sequencing

Full-length genome sequences spanning the entire protein-coding regions were determined for three strains of Psittacine adenovirus HKU1 identified in the present study. Briefly, complete genome sequences of the three strains of Psittacine adenovirus HKU1 were amplified and sequenced directly from the Mealy Parrot samples. Degenerate primers in regions of IVa, polymerase, pIIIa, hexon, 100K and fiber were designed by multiple alignment of genomes of related adenoviruses, including Bovine adenovirus 6 (GenBank accession no. NC_020074), Duck adenovirus A (GenBank accession no. NC_001813) and Ovine adenovirus D (GenBank accession no. NC_004037). Additional primers for subsequent rounds of PCR were designed based on the results of earlier rounds of genome sequencing. These primer sequences are available on request. Sequences for the 5′ and 3′ ends of the viral genome were obtained by random amplification of cDNA ends strategies using the SMARTer RACE cDNA Amplification kit (Clontech). The sequence has been submitted to NCBI GenBank and is available under the accession number KJ675568.

### Phylogenetic tree and sequence analysis

Open reading frames were located using the ORF Finder tool at NCBI (http://www.ncbi.nlm.nih.gov/projects/gorf/), the gene finding program fgenesV0 (http://www.softberry.com/berry.phtml?topic=index&group=programs&subgroup=gfindv), and by comparison with genome annotations of other adenoviruses [20], [21]. Functional annotation of predicted proteins was performed by BLAST similarity search against annotations in RefSeq database and by InterProScan search tool [22]. Multiple alignments of sequences were constructed using MUSCLE [23], and phylogenetic informative regions were extracted using GBLOCKS [24]. Maximum-likelihood phylogenetic trees were constructed using PHYML version 3 [25], under the best-fit protein evolution model as selected by ProtTest 3 [26]. Recombination detection was performed using bootscan analysis in SimPlot [27], with window size of 1000 bp and step size of 100 bp. Whole genome DNA-DNA hybridization values were predicted using Genome-to-Genome Distance Calculator (GGDC) 2.0 [28].

### Viral culture

Viral culture was performed using chick embryo, the avian cell line DF-1, and five other cell lines (HEp-2, Vero E6, human embryonic lung fibroblast, Caco-2 and BSC-1) as previously reported [29], [30]. All infected cell lines were incubated at 37°C for seven days, passaged once and then incubated for another seven days. The cell lines were examined for cytopathic effect using inverted light microscopy on days 1, 3, 5, and 7 of incubation.

### Detection of adenovirus positive cells in tissue specimens

Adenovirus in tissue specimens was detected by immunofluorescence staining. The Psittacine adenovirus HKU1 fiber protein was cloned and expressed to generate specific antibodies. The gene was amplified by primers 5′-GCGAGCTAGCATGTGGCATTTTACTGCAGCCAGT-3′ and 5′-CACTAAGCTTTTATTGTAGTGAAATAAAAGCAGAA-3′. The fiber fragment was then cloned into the *Nhe*I and *Hind*III sites of expression vector pET-28b(+) (Novagen, Madison, Wisconsin) in frame and downstream of a series of six histidine residues. The His6-tagged recombinant fiber protein was expressed and purified by Ni2+-loaded HiTrap chelating system (GE Healthcare, Buckinghamshire, United Kingdom) according to the manufacturer's instructions. Specific guinea pig antiserum was produced by injecting 200 µg of purified His6-tagged recombinant fiber protein, along with an equal volume of complete Freund's adjuvant, into the thighs of guinea pigs intramuscularly. Incomplete Freund's adjuvant was used in subsequent immunizations. Three inoculations were completed in six weeks, with one injection every two weeks. Serum samples were collected two weeks after the last injection. Immunofluorescence staining for adenovirus fiber protein antigen was performed as described previously [30]. The tissue sections were deparaffinized and rehydrated, followed by blocking with 1% bovine serum albumin in PBS to minimize non-specific binding. The sections were incubated overnight with immune serum from guinea pigs at 4°C. After washing three times with PBS, the sections were incubated with FITC-conjugated rabbit anti-guinea pig IgG (Life technology, Invitrogen, Carlsbad, California) at 1∶50 dilution for 30 min at room temperature. The images were captured by Nikon 80i imaging system with Spot Advanced Software. The animal experiment has been approved by the Committee on the Use of Live Animals in Teaching and Research, The University of Hong Kong, in accordance with the Guidelines laid down by the NIH in the USA regarding the care and use of animals for experimental procedures (CULATR 2489-11).

### Statistical analysis

Statistical analysis was performed using GraphPad Prism 6.0. Correlation of *C. psittaci* bacterial load and adenovirus viral load was determined using linear regression analysis. For statistical calculation, 50% of the lower detection limit (6,000 copies/ml for *C. psittaci* and 600 copies/ml for adenovirus) was assigned to specimens that tested negative. Log-transformed viral loads were used for statistical analysis.

## Results

### The index case

The patient was a 55-year-old male artisan working at the NTNAMC. He presented after four days of dyspnea and two days of hemoptysis. Chest radiograph showed bilateral patchy consolidation. Blood test showed neutrophilia and elevated liver enzymes with alanine transaminase of 51 U/L. He was put on non-invasive positive pressure ventilation for respiratory failure. Bronchoalveolar lavage tested positive for *C. psittaci* and rhinovirus by PCR and reverse transcriptase-PCR respectively. Direct fluorescent antigen detection and viral culture did not reveal other viral co-pathogens. Paired serology, collected 18 days apart (on days 2 and 20 after hospitalization), showed a rise of *C. psittaci* IgG titer from <32 to 128 by microimmunofluorescence assay, but there was no increase in adenovirus or other respiratory virus antibody titer. The patient recovered with oral doxycycline. He had contact with birds, monkeys, iguanas and snakes at the NTNAMC within one month of symptom onset.

### The human outbreak

Epidemiological investigation by the Centre for Health Protection of Hong Kong showed that five other members of staff at the NTNAMC were hospitalized for respiratory tract infection, with onset of symptoms from November 6–24, 2012. The details of the five other patients have been reported previously [5].

### Avian chlamydiosis in Mealy Parrots

Sixteen Mealy Parrots imported from Guyana were detained at NTNAMC for observation since October 20, 2012. NTNAMC is a center where animal case-exhibits are detained while pending legal processing. Two Mealy Parrots died naturally, while 14 developed disease and were euthanized. Of the 16 Mealy Parrots, 4 were sent for postmortem examination and 8 were sent for microbiological testing. The 4 remaining Mealy Parrots were not available for further testing.

**Figure 1:**
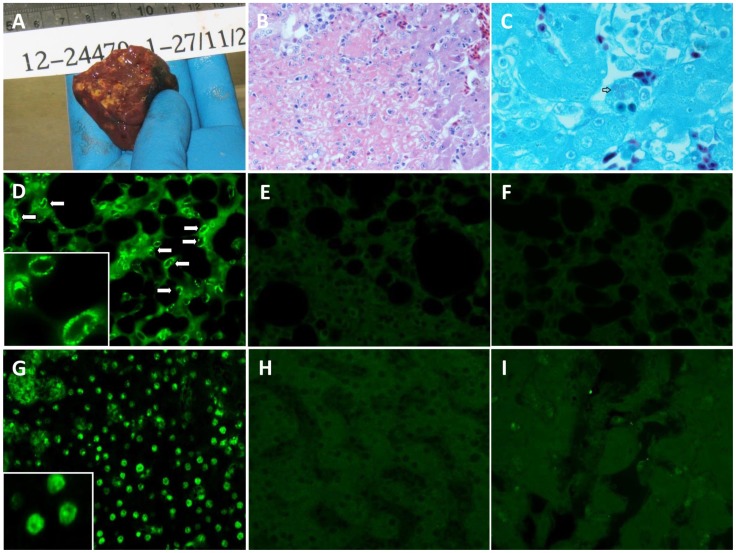
Illustrative histopathological changes of infected birds. Panels A–C: Macroscopic and microscopic changes in the liver of bird 1. Panel A: cut surface of liver showing numerous 1–2 mm coalescing necrosis. Panel B: areas of acute coagulative necrosis, which are randomly distributed throughout the liver (hematoxylin and eosin staining; original magnification, ×400). Panel C: Intracytoplasmic accumulations of Chlamydial organisms (arrow) are commonly found throughout the liver parenchyma (gimenez staining; original magnification, ×1000). Panels D–I: immunochemical staining for Psittacine adenovirus HKU1 fiber protein (Original magnification, ×400). The tissue sections were deparaffinized and rehydrated, followed by blocking with 1% bovine serum albumin in PBS to minimize non-specific binding. The sections were incubated with immune serum (panels D, F, G, I) or with non-immune serum (panels E and H) of guinea pigs. After washing three times with PBS, the sections were incubated with FITC-conjugated rabbit anti-guinea pig IgG. For bird 2, apple green fluorescent foci were seen in the cytoplasm of pneumocytes (D) and in the nucleus of hepatocytes (G) with immune serum. As controls, fluorescent foci were not detected in the lung (E) and liver (H) tissue of bird 2 with non-immune serum. For bird 3, fiber protein-positive cells were not detected in the lung (F) and liver (I) tissue.

Postmortem examination of four Mealy Parrots that were euthanized showed necrotising splenitis in three birds (bird 1, 3, and 4), and necrotising hepatitis in two birds (bird 1 and 4) (Fig. 1A and 1B, Table 2). *C. psittaci* PCR was positive for all four birds, and Giemsa and Gimenez stains for *C. psittaci* were positive in the liver tissue of bird 1 (Fig. 1C).

**Table 2:** Postmortem findings of four parrots obtained during the avian chlamydiosis outbreak.

Bird no.	Gross appearance	Pathology	Specimens tested positive by *Chlamydophila psittaci* PCR
1	Both abdominal air sacs: multiple yellowish plaques of fibrin	Necrotising splenitis	Liver tissue, lung tissue, esophagus tissue, pharyngeal swab, conjunctival swab, cloacal swab
	Liver: numerous pale yellowish foci	Necrotising hepatitis	
	Pericardium: thickened, granular and tightly adherent to the heart	Necrotising nephropathy	
	Spleen: congested and swollen	No significant findings in the lung	
2	Abdominal air sacs: multiple yellowish plaques of fibrin	No significant findings in heart, lung, liver, spleen, small intestine, large intestine, muscle, gizzard and air sac	Esophagus tissue and tracheal swab
	Liver: numerous pale yellowish foci		
	Pericardium: thickened, granular		
3	Left abdominal air sac: cloudy and thickened	Necrotising splenitis	Liver tissue, esophagus tissue, pharyngeal swab and cloacal swab
	Both lungs: greyish pink in color and rubbery to feel	No significant findings in heart, lung, liver, kidney, muscle and trachea	
	Trachea: small amount of yellow caseous material		
	Liver: swollen		
	Pericardium: thickened, granular		
	Peritoneum over the cranial pole of the left kidney: thickened and granular		
	Spleen: enlarged and swollen		
4	Both lungs: greyish pink in color and rubbery to feel	Necrotising splenitis	Liver tissue, pharyngeal swab, tracheal swab, conjunctival swab and cloacal swab
	Liver: swollen	Necrotising hepatitis	
	Spleen: enlarged and swollen	No significant findings in the heart, lung, kidney, trachea, muscle	

Eight other parrots detained during the outbreak were retrieved for microbiological testing (Table 3), in which seven were euthanized because they developed disease and one died. Seven out of eight parrots (87.5%) were positive for *C. psittaci*. All eight parrots were positive for adenovirus by one-step consensus PCR but not the nested PCR. All other viruses were negative by consensus PCR screening. Blood samples were not available from infected parrots for testing. Forty-one additional specimens from 18 healthy parrots unrelated to the outbreak were negative for *C. psittaci* or adenovirus (Table 3). Among parrots, there was a significant association between the presence of adenovirus and *C. psittaci* (*C. psittaci* was present in 7/8 adenovirus-positive parrots vs. 0/18 adenovirus-negative parrots, *P*<0.0001 by Fisher exact test). Moreover, fecal samples taken from 25 other detained mammals, reptiles and avians in NTNAMC during the outbreak were negative for both adenovirus and *C. psittaci*.

**Table 3:** Detection of *Chlamydophila psittaci* and Psittacine adenovirus HKU1 among eight affected parrots and control parrots and animals detained during the outbreak.

		Total no. of specimens available	No. of specimens positive for *C. psittaci*	No. of specimens positive for Psittacine adenovirus HKU1
Affected Mealy Parrots n = 8	Lung	8	4	7
	Kidney	7	6	6
	Liver	8	4	5
	Spleen	5	4	5
	Cloacal swab	8	8	8
Unaffected control parrots* n = 18	All specimens	41	0	0‡
Other detained animals† n = 25	Cloacal/rectal samples	25	0	0‡

*18 healthy parrots, including three Lesser Sulphur-crested Cockatoos (*Cacatua sulphurea*), two Cockatiels (*Nymphicus hollandicus*), two Grey Parrots (*Psittacus erithacus*), two Rose-ringed Parakeets (*Psittacula krameri*), one Greater Sulphur-crested Cockatoo (*Cacatua galerita galerita*), one Red-shouldered Macaw (*Diopsittaca nobilis*), one Monk Parakeet (*Myiopsitta monachus*), one Eclectus Parrot (*Eclectus roratus*), one Salmon-crested Cockatoo (*Cacatua moluccensis*), one Eastern Rosella (*Platycercus eximius)*, one Rosy-faced Lovebird (*Agapornis roseicollis*), one Sun Conure (*Aratinga solstitialis*), and one Blue-and-yellow Macaw (*Ara ararauna*). Cloacal swabs (n = 18), pharyngeal swabs (n = 7) and whole blood samples (n = 16).

† 14 mammals (seven dogs, four cats, three monkeys), seven reptiles (three ball pythons [*Python regius*], two African Spurred Tortoises [*Geochelone sulcata*], two Common Iguanas [*Iguana iguana*]) and four birds (two Grey Parrots, one green peafowl [*Pavo muticus*], one swan).

‡ Consensus nested PCR for adenovirus DNA polymerase was also performed on these specimens, but all were negative.

**Figure 2:**
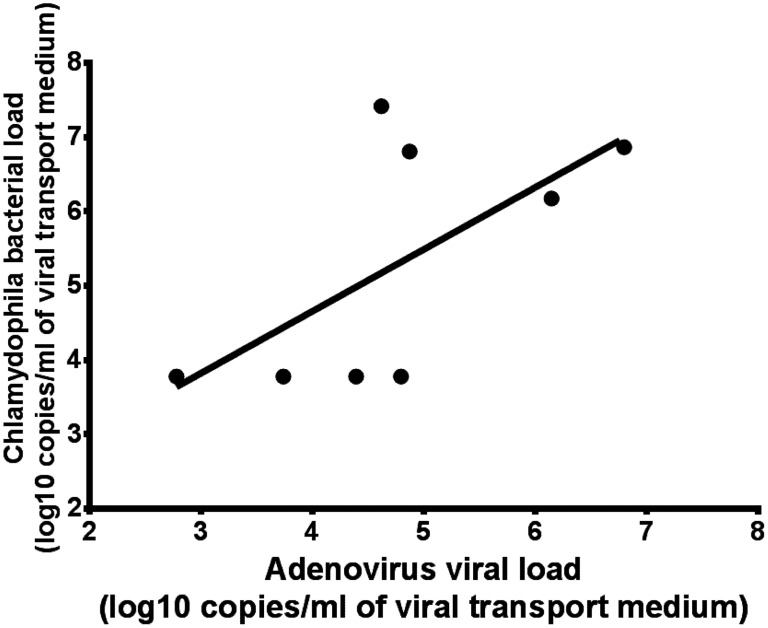
Correlation of adenovirus viral loads and C. psittaci bacterial loads in lung specimens from Mealy Parrots.

Adenovirus viral load testing was performed on tissue and cloacal swab specimens from the infected birds. The *C. psittaci* bacterial load was positively correlated with adenovirus viral load in the lung (r^2^ = 0.4053; *P* = 0.0897) (Fig. 2). Immunofluorescence staining targeting the Psittacine adenovirus HKU1 fiber protein was performed for bird 2 and bird 3. Psittacine adenovirus HKU1 antigen was detected in the lung and liver tissue cells of bird 2 but not bird 3 (Fig. 1D–1I). Viral culture was negative for seven cloacal swabs, three lung samples, two liver samples and two kidney samples, which were positive for adenovirus PCR.

### Screening of the Psittacine adenovirus HKU1 among infected human cases

PCR for the Psittacine adenovirus HKU1 was performed on specimens from four patients, including the whole blood and bronchoalveolar lavage of the index patient, the nasopharyngeal aspirate of two patients, and the sputum of one patient. None of the specimens were positive for this novel avian adenovirus.

### Genome sequencing and analysis of novel Psittacine adenovirus HKU1

**Figure 3:**
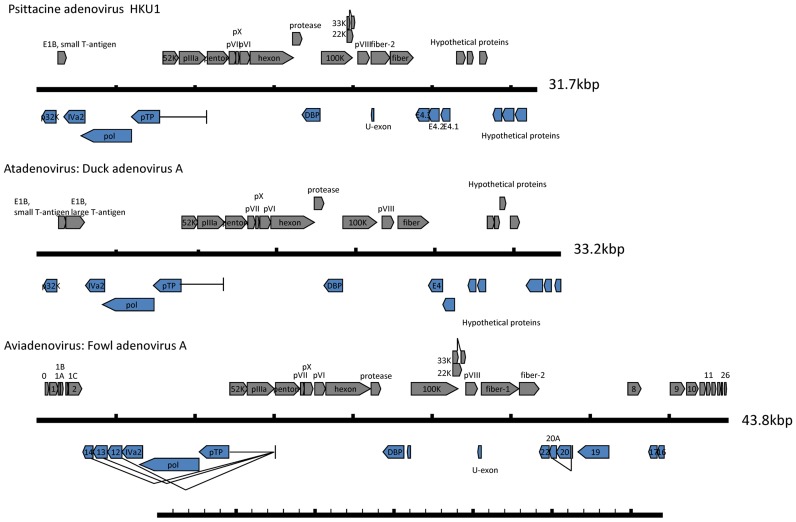
Genome organization of the Psittacine adenovirus HKU1, comparison with duck adenovirus A and fowl adenovirus A.

**Figure 4:**
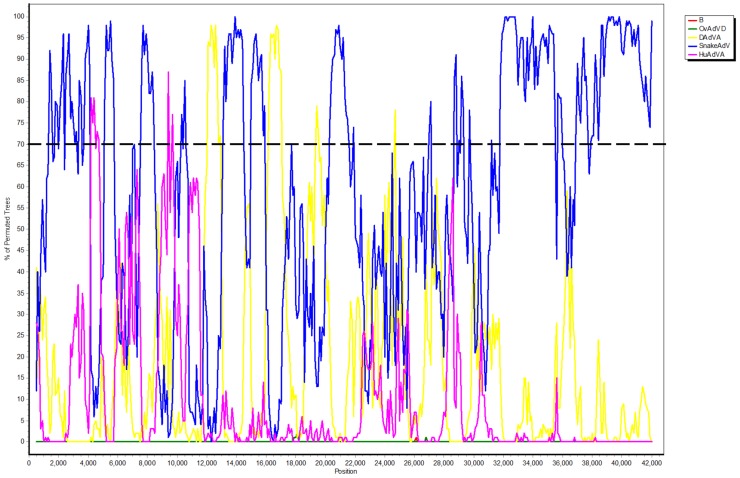
Bootscan analysis using SimPlot did not show strong phylogenetic signal of recombination in the present adenovirus.

Whole viral genome sequencing was performed on three samples positive for adenovirus. Nucleotide sequences of the adenovirus genomes in the three samples were fully identical. The full-length genomic sequence is 31,735 bp in length, with a G+C content of 53.5%. Inverted terminal repeat (ITR) regions of 44 bp at both ends of the genome are complementary to each other. The viral genome contains 23 ORFs with sequence similarity to known adenoviral genes (Fig. 3 and Table 4). Additionally, six ORFs, ranging from 381 to 741 bp were identified at the 3′ end of the genome. While lacking significant sequence similarity to known adenoviral sequences, they were predicted to have coding potential by fgenesV0. The genome organization of this novel virus most closely resembles that of *Atadenovirus*, with minor variations. Similar to Duck adenovirus A, the novel adenovirus lacks LH1, LH2 and LH3, which distinguishes it from the bovine adenovirus E, bovine adenovirus D and ovine adenovirus D. Similar to other *Atadenovirus*, the novel virus possesses a DNA polymerase, terminal protein and DNA-binding protein for DNA replication; the E1B small T-antigen, IVa2 and 52K proteins for DNA encapsidation; and the IIIa, penton protein/III, core protein 1/pVII, core protein 2/pX, pVI protein, hexon protein, endopeptidase/protease, 100K, pVIII protein and fiber protein for the formation and structure of the virion [31]. The novel adenovirus has two fiber proteins. Notably, the fiber-2 protein (or short fiber) is not found in any *Atadenovirus*, but can be found in *Aviadenovirus*. As in other adenoviruses, a putative splicing site was identified in the pTP gene. Recombination analysis by bootscanning showed possible phylogenetic incongruence in small regions of the viral genome (Fig. 4), but further examination by phylogenetic analysis of the corresponding regions did not reveal convincing evidence of recombination.

**Table 4:** Predicted proteins in the Psittacine adenovirus HKU1.

Open reading frame	Gene name	Coding sequence positions		Frame	Size (aa)
		Start	Stop		
1	p32K	1210	293	−3	305
2	E1B, small T-antigen	1177	1713	+1	178
3	IVa2 protein	3033	1702	−1	443
4	DNA polymerase	6005	2772	−2	1077
5	Terminal protein precursor	7777	5978	−3	606
		10540	10520	−1	
6	52K protein	7863	8837	+3	324
7	IIIa protein	8840	10519	+2	559
8	Penton protein/III	10550	11902	+2	450
9	Core protein 1/pVII	11945	12406	+2	153
10	Core protein 2 (mu protein)/pX	12426	12623	+3	65
11	pVI protein	12671	13300	+2	209
12	Hexon protein	13321	16062	+1	913
13	Endopeptidase/protease	16059	16667	+3	202
14	DNA-binding protein	17808	16672	−1	378
15	100K protein	17845	19812	+1	655
16	22K protein	19616	19819	+2	67
17	33K protein	19616	19811	+2	147
		19888	20135	+3	
18	pVIII protein	20144	20896	+2	250
19	U-exon	21073	20909	−3	54
20	Fiber-2 protein	21143	22345	+2	400
21	Fiber protein	22356	23816	+3	486
22	E4.3 protein	24648	23818	−1	276
23	E4.2 protein	25331	24648	−2	227
24	E4.1 protein	25922	25347	−2	191
25	Hypothetical protein	26401	26970	+1	189
26	Hypothetical protein	27242	27622	+2	126
27	Hypothetical protein	28029	28505	+3	158
28	Hypothetical protein	29419	28859	−3	186
29	Hypothetical protein	30185	29445	−2	246
30	Hypothetical protein	30983	30255	−2	242

**Figure 5:**
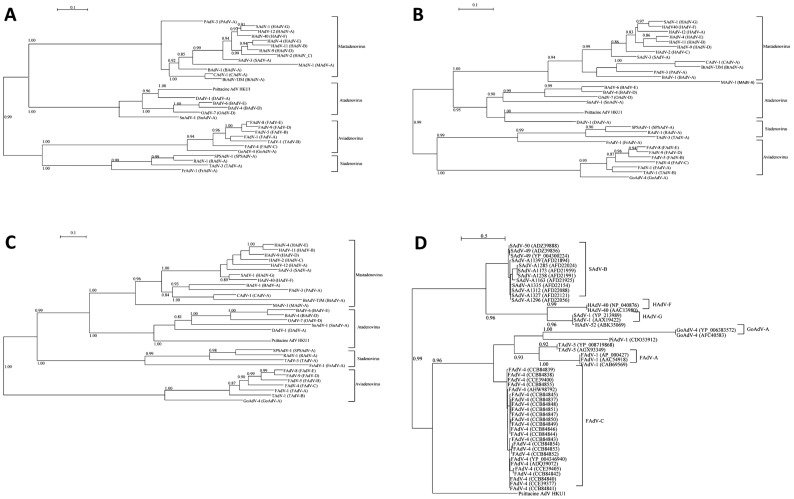
Maximum-likelihood phylogenetic tree showing the relationship of Psittacine adenovirus HKU1 to other adenoviruses inferred from A) hexon protein, B) penton protein and C) polymerase protein, D) fiber-2 protein. The trees were constructed using PHYML version 3 under the best-fit protein evolution model as selected by ProtTest 3. The bootstrap values were calculated from 1,000 trees. (AdV, adenovirus; BAdV, bovine adenovirus; BtAdV, bat adenovirus; CAdV, canine adenovirus; DAdV, duck adenovirus; FAdV, fowl adenovirus; FrAdV, frog adenovirus; GoAdV, goose adenovirus; MAdV, murine adenovirus; OAdV, ovine adenovirus; PAdV; porcine adenovirus; PiAdV, pigeon adenovirus; RAdV, raptor adenovirus; SAdV, simian adenovirus; SnAdV, snake adenovirus; SPSAdV; south polar skua adenovirus; TAdV; turkey adenovirus).

Pairwise amino acid sequence comparison of the major conserved adenoviral genes of this novel adenovirus showed that they were most similar to *Atadenovirus*, with pairwise amino acid identities of 50.3–54.0% for the DNA polymerase, 64.6–70.7% for the penton protein, and 66.1–74.0% for the hexon protein (Table 5). Phylogenetic analysis of the hexon, penton and DNA polymerase amino acid sequences consistently showed clustering of the novel adenovirus with viruses in the genus *Atadenovirus*, most closely related to sequences from Duck adenovirus A (Fig. 5A–5C). The fiber-2 protein amino acid sequence of the novel adenovirus was most closely related to that of Fowl adenovirus C (Fig. 5D). *In silico* DNA-DNA hybridization (DDH) using GGDC 2.0 was also performed to estimate genomic DNA similarity between the novel adenovirus and other adenoviruses. However, the estimated DDH values were at the lower limit of analysis (12.5% for formula 1, and 0% for formulae 2 and 3), which suggested that the genomic DNA sequences are too dissimilar for DDH analysis.

**Table 5:** Pairwise amino acid sequence identities between the novel Psittacine adenovirus HKU1 and other adenoviruses.

	Pairwise amino acid sequence identities (%) to novel Psittacine adenovirus HKU1												
	IVa2	Pol	pTP	52K	IIIa	Penton	pVII	pVI	Hexon	Protease	DBP	100K	pVIII
**Atadenovirus**													
BAdV-4 (BAdV-D)	47.7	51.4	42.1	43.9	46.7	65.2	33.3	47.5	66.1	52.9	40.9	48.7	35.3
DAdV-1 (DAdV-A)	54	54	44.8	55.8	57.6	70.7	59.3	52.1	74	60.3	42.4	48.5	45.9
OAdV-7 (OAdV-D)	47.5	51.9	41.4	41.5	44.1	66.5	29.9	47.1	69.9	51.4	40.8	46.4	35.1
SnAdV-1 (SnAdV-A)	55.4	50.3	39.7	42.3	46.4	66.8	31.2	54	68.8	57.4	39.1	45.4	42.2
BAdV-6 (BAdV-E)	46.6	50.3	42.4	43.3	41.8	64.6	32.2	46.1	68.5	53.9	41	47.5	35.7
**Aviadenovirus**													
FAdV-1 (FAdV-A)	25.7	32.4	26.3	24.8	19.7	45.5	15.4	30	46	40	22	21	15.8
FAdV-5 (FAdV-B)	25	30.6	23.9	22.2	20.4	44.7	16.4	29.9	45.6	40.3	18.8	19.5	15.5
FAdV-4 (FAdV-C)	25.6	31.8	27.5	21.8	19.9	45.2	12	32.4	45.4	36.6	18.6	23.6	14.7
FAdV-9 (FAdV-D)	25.5	31.7	27.3	21.7	18.7	41.7	11.2	31	44.5	38.8	21.6	20.6	15.6
FAdV-8 (FAdV-E)	26.3	32	23.9	21.6	18.9	43.6	14.6	18	45.4	40.4	20.4	19.5	16
GoAdV-4 (GoAdV-A)	25.1	31.5	24.9	22.2	19.7	44.6	16.1	31	46.3	40	16.9	20	15.7
TAdV-1 (TAdV-B)	26.5	30.9	27.3	23	20.2	44.5	15.6	29.7	45.4	37.6	18.2	20.1	15.7
**Siadenovirus**													
FrAdV-1 (FrAdV-A)	24.5	34.6	26.5	22.4	21.3	52.4	10.4	28.6	49.7	40.5	22.1	25.3	17.5
RAdV-1 (RAdV-A)	24.3	37	26.8	18.9	22.3	51.7	13.1	30	49.7	41	20.6	26.9	18.6
SPSAdV-1 (SPSAdV-A)	24.5	37	25.2	17.9	22.4	51.5	11.6	30	47.5	42.5	22.1	27.8	18.4
TAdV-3 (TAdV-A)	24.1	36.3	26.3	18.6	21.4	49.4	11.1	29.8	49.5	38	21.1	27.6	15.7
**Mastadenovirus**													
BtAdV-TJM (BtAdV-A)	32.4	35.7	25.8	18.9	27.2	45.1	22.8	15.8	52	39.2	19.1	24.8	18.9
BAdV-1 (BAdV-A)	33.9	38.4	25.5	21.9	26.4	52.9	18.1	12.3	52.5	37.9	24.2	25.5	18.5
CAdV-1 (CAdV-A)	32.8	39.5	25.2	20.2	27.1	48.4	22.8	16	52.3	38.2	20.8	25.4	18.4
HAdV-12 (HAdV-A)	33.1	37.4	25.9	19.8	27.5	48.1	25.2	15.2	49.4	40	17.6	25.1	19.7
HAdV-11 (HAdV-B)	32.6	37.6	25.9	19.7	28	43.9	24.2	16.6	47.7	38.5	17.9	24.1	20
HAdV-2 (HAdV-C)	32.8	37.4	25	18.7	26.6	42.8	23.5	16.1	47.9	38.8	17.7	23.9	20
HAdV-9 (HAdV-D)	32.6	37.7	25.9	20.3	27.8	47.4	23.5	16	48.3	38.5	19.9	26	19.3
HAdV-4 (HAdV-E)	32.6	37.3	26.2	20.2	27.2	46.2	25.2	16	48.6	39.6	18.7	25.6	20
HAdV-40 (HAdV-F)	32.4	37.4	26.2	21.3	27.5	48.4	23.9	15.7	49.5	39.8	20.5	25.4	19.7
SAdV-1 (HAdV-G)	32.6	37.7	26.5	21	27	48.7	23.1	15.2	49	40.7	18.6	26.6	20.4
MAdV-1 (MAdV-A)	35.6	36.8	23.4	21.5	24	45.8	23.1	17.2	50.9	43.6	20.6	25.7	19.4
PAdV-3 (PAdV-A)	32.4	37.5	26.9	19.8	24.3	51.7	21.6	18.1	50.9	38.3	21.3	23.1	18.1
SAdV-3 (SAdV-A)	32.6	38	26.5	19.7	26.3	48.5	23.8	13.9	49	39.3	20.4	25.3	21.5

## Discussion

To the best of our knowledge, this is the first report of a co-infection of psittacine birds with avian adenovirus and *C. psittaci* associated with an outbreak of human psittacosis. In this study, we have identified a novel adenovirus that was most closely related to Duck adenovirus A of the *Atadenovirus* genus in the epidemiologically linked Mealy Parrots. In contrast, this novel adenovirus was not identified in any of the healthy parrots and other detained animals without *C. psittaci* infection. Psittacine adenovirus HKU1 antigen was detected in lung and liver tissue cells using immunostaining, which indicated active viral replication instead of latent infection. A positive correlation between adenovirus viral loads with *C. psittaci* bacterial loads was observed in lung specimens, which suggested a possible synergistic interaction between adenovirus and *C. psittaci* in disease pathogenesis.

In birds, many adenoviral infections are subclinical, but some infections can lead to severe disease [32], [33]. Previous studies have suggested that avian adenoviruses may cause immunosuppression in birds. *Aviadenovirus* can lead to immunosuppressive disease, such as the hydropericardium syndrome. Fowl adenovirus and chicken anemia virus co-infection causes much more severe disease than either virus alone [34]. Avian adenoviruses have been shown to directly infect lymphocytes and dendritic cells in the spleen [35] and causes depletion of lymphocytes [36], [37]. Fowl adenovirus 1 has been shown to affect antibody response of chicks to *Brucella abortus* [38]. We speculate that our novel adenovirus may have caused immunosuppression among the infected parrots, and therefore a larger number of Mealy Parrots were infected by *C. psittaci* with a higher bacterial load, leading to a higher chance of zoonotic transmission to humans. Other studies have also proposed that reovirus and avian pneumovirus infection may cause immunosuppression leading to avian chlamydiosis [11], [12]. Experimental infection showed that avian pneumovirus could exacerbate acute *C. psittaci* infection in turkeys [12]. Alternatively, in a report demonstrating adenovirus-*C. psittaci* co-infection in a parakeet using electron microscopy and antigen detection without molecular confirmation, Desmidt M *et al* proposed that *C. psittaci* could cause immunosuppression, which can lead to reactivation of latent adenovirus infection [10]. However, no further studies have been performed to verify this hypothesis.

In psittacine birds, adenovirus infection manifests as depression, anorexia, diarrhea and cloacal hemorrhage [39]. Gross examination may show hepatomegaly, splenomegaly, dilatation of the duodenum and proventriculus, swollen kidneys, and edema, congestion, and hemorrhage of the lungs. Histological changes may include necrosis in the liver and the spleen. A case of inclusion body hepatitis has been described [40]. The pathological features in our infected parrots were typical of *C. psittaci* infection, but some of these are actually indistinguishable from adenovirus infection as one Mealy Parrot was also positive for the Psittacine adenovirus HKU1 on immunofluorescence staining.

Current ICTV guidance on adenovirus taxonomy specifies species designation by phylogenetic distance, host range, DNA hybridization, nucleotide composition, cross-neutralization and gene organization at the right end of the viral genome [41]. With the exception of nucleotide composition and cross-neutralization, for which no data are available, our data and analysis of the Psittacine adenovirus HKU1 are consistent with it being a novel member of the genus *Atadenovirus*. Notably, the genome of the Psittacine adenovirus HKU1 has a higher G+C content than other *Atadenovirus* genomes, which might be viewed as a feature against its designation as a novel *Atadenovirus*. Indeed, the high A+T content of the first few identified *Atadenoviruses* was considered characteristic among adenoviruses and was the basis for naming the genus. Nonetheless, the snake adenovirus 1, a member of the *Atadenovirus*, has a genomic G+C content of 50.2%, which is lower than but comparable to the G+C content of 53.5% for the Psittacine adenovirus HKU1. Hence, we consider that the high genomic G+C content of the present Psittacine adenovirus HKU1 is not a major contraindication to its inclusion in the genus *Atadenovirus*.

Besides the genomic G+C content, there are several unusual genomic features in the Psittacine adenovirus HKU1. Firstly, a second viral protein, fiber-2, is present in our novel adenovirus, but not in any other *Atadenovirus*. The fiber-2 protein has also been found in the *Aviadenovirus*, like the fowl adenovirus. Since fiber proteins are responsible for binding to cellular receptor, it has been postulated that the presence of different fiber proteins may determine the tropism of the adenovirus [42]. It remains to be determined whether the fiber-2 protein is important for the virus to infect psittacine hosts. Another interesting feature is the predicted CCU start codon of the IVa2 protein. The annotation is made on the basis of sequence conservation among other adenoviral IVa2 proteins, and the lack of an in-frame ATG in its vicinity. The function of the non-canonical CCU start codon may be augmented by the presence of an upstream Kozak sequence ACCACC. The use of CCU as the start codon is rare in other organisms, but has been reported in psittacine viruses. The other notable genomic feature in the Psittacine adenovirus HKU1 is the intron between the two exons of 33K, which usually overlaps with coding sequences in other adenoviruses, but not in the present case.

Virus and bacteria often act synergistically in causing diseases in humans or animals. *C. psittaci*-avian pneumovirus co-infection has been associated with an outbreak in turkeys [12], *C. psittaci*-fowlpox virus co-infection with an outbreak in hens [9] and *C. psittaci*-reovirus with an outbreak in budgerigars [11]. Infectious bursal disease virus and chicken anaemia virus can cause immunosuppression, leading to secondary bacterial infection such as bacterial chondronecrosis with osteomyelitis [43]. However, it is unclear whether co-infection in birds can increase the risk of transmission of avian pathogens to humans. Our investigation suggested that the novel Psittacine adenovirus may have been associated with immunosuppression among infected birds, leading to higher *C. psittaci* bacterial loads in the lungs of psittacine birds, and hence increasing the risk of infection in exposed humans.
